# Astrocytic Calcium Dynamics Along the Pain Pathway

**DOI:** 10.3389/fncel.2020.594216

**Published:** 2020-10-16

**Authors:** Jeiwon Cho, Yeowool Huh

**Affiliations:** ^1^Brain and Cognitive Science, Scranton College, Ewha Womans University, Seoul, South Korea; ^2^Department of Medical Science, College of Medicine, Catholic Kwandong University, Incheon, South Korea; ^3^Translational Brain Research Center, Catholic Kwandong University, International St. Mary’s Hospital, Incheon, South Korea

**Keywords:** astrocyte, calcium, calcium channels, pain pathway, chronic pain, neuropathic pain, inflammatory pain

## Abstract

Astrocytes, once thought to be passive cells merely filling the space between neurons in the nervous system, are receiving attention as active modulators of the brain and spinal cord physiology by providing nutrients, maintaining homeostasis, and modulating synaptic transmission. Accumulating evidence indicates that astrocytes are critically involved in chronic pain regulation. Injury induces astrocytes to become reactive, and recent studies suggest that reactive astrocytes can have either neuroprotective or neurodegenerative effects. While the exact mechanisms underlying the transition from resting astrocytes to reactive astrocytes remain unknown, astrocytic calcium increase, coordinated by inflammatory molecules, has been suggested to trigger this transition. In this mini review article, we will discuss the roles of astrocytic calcium, channels contributing to calcium dynamics in astrocytes, astrocyte activations along the pain pathway, and possible relationships between astrocytic calcium dynamics and chronic pain.

## Introduction

Astrocytes play critical roles in the central nervous system (CNS). Under physiological conditions, astrocytes occupy non-overlapping regions and function in discrete areas (Nedergaard et al., 2003). Nonetheless, astrocytes are joined to adjacent astrocytes by gap junctions (Dermietzel et al., 1991), which make them apt for coordinating activity. Although several types of voltage-gated ion channels have been reported in astrocytes, there are not enough voltage-gated Na^+^ channels expressed to generate action potentials; thus, astrocytes are not considered electrically excitable (Pappalardo et al., 2016). However, astrocytes are reported to respond to external stimuli with internal Ca^2+^ elevation (Wang et al., 2006; Agulhon et al., 2008; Rusakov, 2015; Bazargani and Attwell, 2016; Guerra-Gomes et al., 2017), and this suggests that Ca^2+^ dynamics are an important signaling mechanism in astrocytes. Furthermore, a stimulated astrocyte with increased intracellular Ca^2+^ could subsequently trigger intracellular Ca^2+^ rise in non-stimulated astrocytes and propagate Ca^2+^ signals, a phenomenon called an astrocytic Ca^2+^ wave (Cornell-Bell et al., 1990; Dani et al., 1992; Charles, 1998; Scemes and Giaume, 2006). Astrocytic Ca^2+^ waves enable astrocytes to communicate over long distances. It is now generally accepted that astrocytic Ca^2+^ waves usually do not occur in physiological conditions (Agulhon et al., 2008). Accordingly, astrocytic Ca^2+^ waves are commonly observed after injury or in pathological conditions. Whether Ca^2+^ signal propagation between astrocytes in these conditions provides protective or detrimental effect is not clear-cut. It is, however, clear that the Ca^2+^ dynamics of astrocytes change in several pathological conditions, including neuropathic pain (Shigetomi et al., 2019). Therefore, understanding changes in astrocytic Ca^2+^ signaling related to pathological pain may provide insight into developing better methods for pain control. In this review, we delved to provide an overview of the relationship between astrocytic Ca^2+^ and chronic pain.

## Roles of Astrocytic Calcium

Astrocytic Ca^2+^ has been implicated in various supportive and active roles within the CNS, from regulating synaptic transmission to modifying behavior.

At the synaptic level, astrocytes provide many supportive roles that modulate synaptic transmission between neurons. One way astrocytes regulate neuronal excitability is by buffering extracellular ions and maintaining optimal concentrations. Clearing increased K^+^ from the extracellular space is vital for limiting the hyper-excitability of neurons, and dysfunction in astrocytic K^+^ buffering capability underlies several neurological diseases such as epilepsy and Huntington’s disease (Bellot-Saez et al., 2017). K^+^ uptake from the extracellular space by astrocyte results in astrocytic Ca^2+^ dependently (Quandt and MacVicar, 1986; Wang et al., 2012), showing the importance of astrocytic Ca^2+^ in regulating K^+^ homeostasis.

Astrocytes also play a key role in buffering neurotransmitters, which is in part regulated by astrocytic Ca^2+^. A single astrocyte can cover both excitatory and inhibitory neurons and buffer both neurotransmitters: excitatory glutamate (Glu) and inhibitory γ-aminobutyric acid (GABA). The majority of Glu in the extracellular space is absorbed (80–90%) by excitatory amino-acid transporter (EAAT)-1 and 2, which are predominantly expressed in astrocytes (Mahmoud et al., 2019). Whether astrocytic Ca^2+^ regulates the activity of EAAT is unknown, but EAAT activity raises intracellular Ca^2+^ in astrocytes (Mahmoud et al., 2019), which would lead to subsequent Ca^2+^ signaling cascades. GABA transporter (GAT) activity, on the other hand, is directly regulated by astrocytic Ca^2+^. Transient receptor potential (TRP) channel subfamily A (TRPA), a cation channel shown to be important in maintaining basal astrocytic Ca^2+^ concentration in astrocytes, controls the expression of GAT in a Ca^2+^-dependent manner (Shigetomi et al., 2011). In addition to basal Ca^2+^ concentration, changes in astrocytic Ca^2+^, such as Ca^2+^ influx into astrocytes or Ca^2+^ reduction in astrocytes, influence the activity and functional expression of GAT in astrocytes (Zhang et al., 2017; Yu et al., 2018). Both types of transporters, EAAT and GAT, are populated around astrocytic processes that wrap around synapses (Minelli et al., 1996; Voutsinos-Porche et al., 2003), making them ideal for buffering and maintaining an appropriate level of neurotransmitters. Accordingly, astrocytic Ca^2+^ has major supportive roles in maintaining an optimal level of excitatory and inhibitory balance of neurons.

Perisynaptic astrocytic process (PAP), a structure of fine astrocytic processes wrapped around neuronal synapses, affect astrocytic buffering ability and provide spine stability in an astrocytic Ca^2+^-dependent manner (Bernardinelli et al., 2014). PAPs are extremely fine structures on the scale of nanometers, and the extent of area covered by PAPs affects astrocytic neurotransmitter uptake, with more coverage leading to more neurotransmitter uptake (Pannasch et al., 2014). Uncaging astrocytic Ca^2+^ induced actin-dependent outgrowth of PAPs, whereas reduced Ca^2+^ signaling in astrocytes reduced synaptic coverage by astrocytes (Bernardinelli et al., 2014). Furthermore, *in vivo* whisker stimulation, which increases neuronal activity in the somatosensory cortex and elevates astrocytic Ca^2+^, also enhances the movement of PAPs, demonstrating that astrocytic Ca^2+^ has physiological roles *in vivo* (Bernardinelli et al., 2014).

Another way astrocytes support CNS function is by providing nutrients and oxygen for metabolism. Lactate, the main nutrient secreted by astrocytes, enables hippocampal long-term potentiation (LTP) between synapses (Suzuki et al., 2011), which is hypothesized to be the cellular basis for learning and memory. In accordance, specifically inhibiting lactate secretion from astrocytes in the hippocampus disrupted hippocampal-dependent learning and memory in mice (Suzuki et al., 2011). Whether Ca^2+^ affects astrocytic lactate secretion is unknown, but the control of vascular smooth muscle tone, which is another important way of providing nutrients and oxygen, is directly regulated by astrocytic Ca^2+^ (Winship et al., 2007; Otsu et al., 2015). Sensory stimulations were shown to trigger rapid astrocytic Ca^2+^ responses to regulate neurovascular coupling (Stobart et al., 2018a).

Astrocytes are also proposed to actively modulate synaptic signal transmission by releasing gliotransmitters, as part of the tripartite synapse (Halassa et al., 2007; Santello et al., 2012). Major gliotransmitters released by astrocytes are ATP, D-serine, GABA, and Glu. Whether Ca^2+^-dependent gliotransmitter release occurs in physiological conditions remains unresolved with debates for and against it (Fiacco and McCarthy, 2018; Savtchouk and Volterra, 2018). Opponents argue that evidence for Ca^2+^-dependent gliotransmitter release comes from cultured astrocytes or acute brain slice where tissue damage is inevitable (Agulhon et al., 2012; Fiacco and McCarthy, 2018). Inflammatory mediators such as tumor necrosis factor-α (TNF-α) and activated microglia resulting from tissue damage will transform resting astrocytes into reactive astrocytes, and reactive astrocytes are well-known to show astrocytic Ca^2+^-dependent gliotransmitter release. However, these cases do not negate Ca^2+^-independent gliotransmitter release, mediated by channels or transporters (Savtchouk and Volterra, 2018), and although inconclusive, Ca^2+^-dependent gliotransmitter release may also be present under physiological conditions.

Astrocytes, under physiological conditions, may or may not release gliotransmitters Ca^2+^ dependently, but many studies suggest that astrocytic Ca^2+^ clearly has physiological functions. Natural stimulations such as visual or whisker stimulation elicit astrocytic Ca^2+^ responses *in vivo* and modulation of astrocytic Ca^2+^ changed behavior (Wang et al., 2006; Stobart et al., 2018b). Astrocytic Ca^2+^ activity was found to be an important regulator of cortical oscillation switches *in vivo* (Poskanzer and Yuste, 2016). Various behaviors such as learning and memory, sleep patterns, and repetitive behaviors were altered by modulations of astrocytic Ca^2+^ (Srinivasan et al., 2015; Adamsky et al., 2018; Stobart et al., 2018b; Yu et al., 2018). Behaviors resembling neuropathic pain were also induced by selective optogenetic activation of astrocytes, which increased astrocytic Ca^2+^ in the spinal cord (Nam et al., 2016). Spontaneous astrocytic Ca^2+^ oscillation is also suggested to be important in generating brain rhythms (Buskila et al., 2019), and a human study suggested that alterations in Ca^2+^ could cause chronic pain (Alshelh et al., 2016, 2019).

As described above, astrocytics Ca^2+^ signals are crucial for maintaining CNS function. Astrocytic Ca^2+^ signals may not be as fast as neuronal signals (second vs. millisecond range, respectively), but they could be more appropriate for providing sustained signaling in cases such as protecting tissues after an injury during recovery. While some alterations in astrocytic Ca^2+^ signals have been associated with dysfunctions in the CNS such as chronic pain, what kind of astrocytic Ca^2+^ changes lead to pathological conditions is still unclear. Since astrocytic Ca^2+^ signals triggered by different sources seem to have different functions, different sources of astrocytic Ca^2+^ are discussed in the next section.

## Source of Calcium in Astrocytes

Several Ca^2+^ sources contribute to astrocytic Ca^2+^ dynamics. Ca^2+^ releases from intracellular stores and influx from the extracellular space both contribute to astrocytic Ca^2+^ changes, albeit with different patterns. Intracellular sources of astrocytic Ca^2+^ include inositol triphosphate receptor (IP3R), ryanodine receptor (RyR), and mitochondrial permeability transition pore (mPTP). Ca^2+^ Channels that contribute to Ca^2+^ influx into astrocytes from the extracellular space include Orai channels, ligand-gated ion channels (AMPA, NMDA, P2X, nicotinic acetylcholine, and 5-HT3 receptors), TRP channels, sodium/calcium exchangers (NCX), and voltage-gated Ca^2+^ channels.

The major source of astrocytic Ca^2+^ increase occurs by activation of IP3R expressed on the endoplasmic reticulum (ER). Activation of IP3R leads to a robust Ca^2+^ increase in the cell body. Ca^2+^ dynamics become nearly absent in the cell body of astrocytes with inhibition of IP3R or knockout of IP3R2, a major type of IP3R in astrocytes, but some Ca^2+^ activity in astrocytic processes remains (Srinivasan et al., 2015). The remaining Ca^2+^ activity in astrocytic processes could be from mPTP (Agarwal et al., 2017), RyR (Matyash et al., 2002), or influx from extracellular space *via* various channels and exchangers (Rungta et al., 2016). Astrocytic IP3R may be targeted to regulate chronic pain since direct and indirect reductions of IP3R activity were correlated with reductions in pathological pain symptoms associated with neuropathic pain in mice (Kim et al., 2016; Ishikawa et al., 2018).

RyR, another type of calcium channel expressed on the ER, is another intracellular source of astrocytic Ca^2+^. Although not a major contributor to astrocytic Ca^2+^ in hippocampal slices (Beck et al., 2004), it is reported to have supportive roles in generating Ca^2+^ oscillation in ventrobasal thalamic slices (Parri and Crunelli, 2003). RyR may also contribute to recovery after an injury, since RyR3 was shown to control astrocyte motility, an important component of healing and regeneration after brain injury (Matyash et al., 2002).

mPTP, a non-selective Ca^2+^-permeable channel expressed on mitochondria, is another possible contributor of intracellular Ca^2+^ increase in astrocytes. Unlike the IP3R, which triggers strong Ca^2+^ increases throughout the cell body, mPTP was shown to be responsible for local Ca^2+^ changes in astrocytic processes (Agarwal et al., 2017). Mitochondria located in astrocytic processes will provide energy and Ca^2+^ signals in microdomains under physiological conditions. However, in the presence of reactive oxygen species (ROS) generated by mitochondria and the increase in Ca^2+^ released from the ER, the mitochondrial membrane depolarizes *via* the opening of the mPTP, which ultimately leads to the necrotic death of astrocytes (Jacobson and Duchen, 2002). Both ROS and ER Ca^2+^ were required for this event to occur, and the transient opening of mPTP itself was innocuous, leading to no changes in apoptosis nor necrosis. These studies suggest that mPTP contributes to cellular signaling in physiological conditions and contributes to necrosis in the presence of ROS and ER Ca^2+^ release.

The major route of Ca^2+^ influx into astrocyte from extracellular space is by Orai channels. Orai channels are store-operated channels regulated by intracellular Ca^2+^ stores such as the ER (Smyth et al., 2006; Hewavitharana et al., 2007). When Ca^2+^ in the ER is depleted, stromal interaction molecules (STIMs) on the ER, which sense Ca^2+^, cluster to activate Orai channels (Zhang et al., 2005; Wang et al., 2009). Activated Orai channels not only replenish internal Ca^2+^ stores but also induce Ca^2+^-dependent gliotransmitter release and regulate inhibition by increasing GABAergic interneuron activity (Toth et al., 2019). Of the Orai channels expressed in astrocytes (Orai 1/2/3), activation of Orai1 was shown to promote the production of inflammatory cytokines in spinal astrocytes (Gao et al., 2016). Orai1 is also upregulated with neuroinflammation induced by lipopolysaccharide (LPS; Wei et al., 2019), suggesting that Orai1 could contribute to increased astrocytic Ca^2+^ following inflammation.

Several types of ligand-gated non-selective cation channels are also sources of Ca^2+^ elevation in astrocytes. ATP-gated purinergic receptor P2X and inotropic glutamate receptors such as AMPA and NMDA receptors are found in astrocytes. P2X_7_ could contribute to Ca^2+^ influx into astrocytes because a marked increase in astrocytic Ca^2+^ is observed with a P2X_7_ agonist application, but no changes were observed with a P2X_1_/P2X_3_ agonist application (Fumagalli et al., 2003). Most AMPA receptors are Ca^2+^ impermeable, but astrocytes in some brain regions, such as those expressed in olfactory astrocytes or Bergmann glia in the cerebellum, express GluA2 lacking AMPA receptors, which are Ca^2+^ permeable (Iino et al., 2001; Matthias et al., 2003). NMDA receptors are also expressed in some brain regions such as the neocortex, but not in the hippocampus, and they contribute to the regional heterogeneity of Ca^2+^ influx (Dzamba et al., 2013). These regional differences in expression patterns will likely contribute to different roles of astrocytes played in each region.

TRP channels are also an important source of astrocytic Ca^2+^ influx. TRPA1 has high Ca^2+^ conductance and has been shown to be an important regulator of resting Ca^2+^ concentration in astrocytes. Blocking TRPA1 not only reduced astrocytic Ca^2+^ concentration, but it also reduced the efficiency of inhibitory synapses by reducing GAT-3 activity in astrocytes (Shigetomi et al., 2013). TRPV1 and TRPV4 are also Ca^2+^-permeable channels expressed in astrocytes. Ca^2+^ entry through TRPV1 channel, but not Ca^2+^ entry through P2X_4_, induced strong Ca^2+^-dependent inactivation of Orai channels, thereby reducing Orai mediated Ca^2+^ entry, which also reduced wound healing measured by scratch-wound assay. This suggests that astrocytic Ca^2+^ signals induced by different channels would have different functions (Bastian-Eugenio et al., 2019). TRPV4 expression was also shown to be upregulated in ischemia, and it may be one underlying factor of astrocytic Ca^2+^ elevation following middle cerebral artery occlusion (Rakers et al., 2017). TRPC is another contributor to the astrocytic Ca^2+^ influx (Belkacemi et al., 2017). TRPC is a stretch-activated channel that mediates store-operated Ca^2+^ entry, similar to Orai channels. Orai and TRPC form complexes and participate in Ca^2+^ entry into astrocytes (Liao et al., 2009; Verkhratsky et al., 2014). However, the activation mechanism of the two channels slightly differs: Orai is STIM dependent, while TRPC could be either STIM dependent or independent. TRPC also has been implicated in the gliotransmitter release, showing that blocking TRPC1 reduced mechanically induced Ca^2+^-dependent Glu release (Malarkey et al., 2008). In the TRC3 knockout mice, TRPC3 mediated Ca^2+^ entry into astrocytes, and injury-induced astrogliosis was reduced (Belkacemi et al., 2017).

NCX working in reverse mode could also elevate astrocytic Ca^2+^. NCX usually works in a forward mode, extruding Ca^2+^ and transporting Na^+^ into the cell. When intracellular Na^+^ concentration increases, NCX extrudes Na^+^ and brings Ca^2+^ in Brazhe et al. (2018) and Wade et al. (2019). Several mechanisms are reported to increase intracellular Na^+^ concentration in astrocytes. NMDA mediated Na^+^ influx into neocortical astrocytes drive Ca^2+^ influx through NCX (Rose et al., 2020). Neurotransmitter uptake mediated by transporters such as GLT-1 or GAT-3 increase Na^2+^ in astrocytes, driving NCX to operate in reverse mode (Bazargani and Attwell, 2016). NCX working in reverse mode could be an important source of Ca^2+^ elevation in fine astrocytic processes, such as at PAPs that ensheath synapse. NCX was found to be enriched in PAPs, and the extremely fine nanometer volume of PAPs impedes the diffusion of Ca^2+^ (Rusakov et al., 2011; Rusakov, 2015). ER and mitochondria, which are intracellular sources of Ca^2+^, are absent in the fine astrocytic process likely due to physical constraints. Therefore, it is reasonable to assume that Ca^2+^ activity in fine astrocytic processes is segregated from Ca^2+^ activity in the soma or thick processes and that the major source of Ca^2+^ increase in fine astrocytic processes is an influx from the extracellular space.

Voltage-gated Ca^2+^ channels (VGCCs) could also contribute to astrocytic Ca^2+^ elevation. Several types of voltage-gated Ca^2+^ channels—L, N, R, and T types—are expressed in astrocytes. Although astrocytes are not electrically excitable, cultured astrocytes responded with an increase in intracellular Ca^2+^ to electrical stimulation (Latour et al., 2003; D’Ascenzo et al., 2004). Astrocytic Ca^2+^ changes mediated by VGCCs were also reported in slice experiments in the subventricular zone and ventrobasal thalamus (Parri et al., 2001; Parri and Crunelli, 2003; Young et al., 2010). Whether these channels are functional in resting astrocytes is unclear (Carmignoto et al., 1998), but L-type Ca^2+^ channel in astrocytes was significantly upregulated with inflammation or injury and contribute to the consequent increase in astrocytic Ca^2+^ (Westenbroek et al., 1998; Cheli et al., 2016). Blocking L- and N-type Ca^2+^ channel antagonist reduced the death of neurons and astrocytes (Gurkoff et al., 2013), and specific knockdown/knockout of L-type Ca^2+^ channels (Ca_v_1.2) in astrocytes prevented astrocyte activation, reduced inflammation in the brain, and promoted myelin regeneration (Zamora et al., 2020), suggesting that astrocytic VGCCs could be therapeutic targets to reduce inflammation and promote recovery.

As described above, various channels lead to different Ca^2+^ dynamics in astrocytes. An increase in astrocytic Ca^2+^ is not a uniform process. Rather Ca^2+^ increases mediated by different channels having diverse consequences. Astrocyte diversity among different brain regions has been well characterized (Matias et al., 2019). Even within the same brain region, diverse populations of astrocytes with different functions have been reported (Yu et al., 2018). Sexual dimorphism of astrocytes has also been reported (Chen et al., 2018), and this could be one of the factors underlying the difference in pain prevalence between males and females. Therefore, the multi-faceted sources and consequence of astrocytic Ca^2+^ dynamics should be considered when investigating astrocyte physiology.

## Astrocyte Activation Along the Pain Pathway With Chronic Pain

Astrocytes react to noxious stimulation and injury and become reactive astrocytes. There are mixed opinions on what defines a reactive astrocyte (Escartin et al., 2019), but reactive astrocytes are often characterized by morphological changes (hypertrophy), proliferation, gene expression changes, receptor changes, and functional changes. Functions of reactive astrocytes are completely different from astrocytes in a non-reactive state called resting astrocytes. Resting astrocytes have been suggested to attenuate pain, while reactive astrocytes have been suggested to contribute to chronic pain (Ji et al., 2019). Chronic pain discussed in this review will mainly include long-lasting pathological pain caused by inflammation or nerve damage. Dual effects of reactive astrocytes are gaining attention with the finding of two different types of reactive astrocytes named the A1 and A2, which have neurodegenerative and neuroprotective effects, respectively (Liddelow and Barres, 2017; Liddelow et al., 2017). Complement component 3 (C3) is the most highly upregulated gene in the A1 subtype, while the S100A10 was pointed out to be A2 subtype specific (Liddelow et al., 2017). Although the presence of A1 and A2 reactive astrocytes has been identified, whether they play different roles in chronic pain is uninvestigated. It is speculated, however, that the A1 subtype would cause chronic pain while the A2 subtype will reduce chronic pain (Li et al., 2019). Reactive astrocytes have been suggested as one of the factors contributing to the maintenance of chronic pain. Expression of glial fibrillary acidic protein (GFAP), a common marker of reactive astrocytes, was enhanced for 9 months in rats following spinal nerve injury (Gwak et al., 2012). Studies showing that inhibition of GFAP expression reduces neuropathic pain behaviors suggest that GFAP upregulation could lead to chronic pain (Ji et al., 2013). Moreover, injecting reactive astrocytes into the spinal cord induced mechanical allodynia in naïve mice, while drugs inhibiting astrocyte activation reversed mechanical allodynia (Gao et al., 2010), strongly supporting that reactive astrocytes contribute to triggering and maintaining chronic pain symptoms. These studies suggest a potential causative relationship between GFAP upregulation in reactive astrocytes and chronic pain.

**Figure 1:**
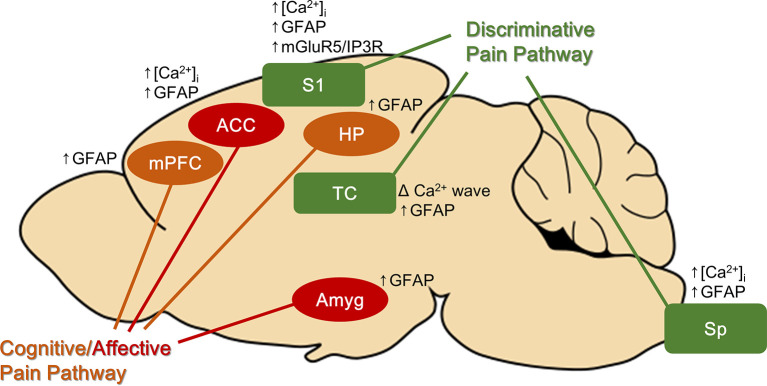
Brain areas found to have reactive astrocytes with chronic pain. Astrocytes in both discriminative and cognitive/affective pain pathways are activated with chronic pain. Some of the brain regions have astrocytic Ca^2+^ signal changes. ACC, anterior cingulate cortex; Amyg, amygdala; HP, hippocampus; mPFC, medial prefrontal cortex; S1, primary somatosensory cortex; Sp, spinal cord.

Pain has both sensory discriminative (location and intensity) and affective (emotional unpleasantness) aspects, which are separately processed by discriminatory and the affective pain pathways. Interestingly, in chronic pain models, reactive astrocytes are found not only at the site of noxious stimulation or injury but also along both pain pathways (Figure 1). Astrocytes were activated in brain regions associated with the discriminative aspect of pain (the spinal cord, thalamus, and primary sensory cortex) and with the affective-cognitive aspect of pain [the anterior cingulate cortex (ACC), amygdala, medial prefrontal cortex, and hippocampus] (to be discussed in subsequent paragraphs). Similar glial activation patterns were reported in a human *in vivo* study, suggesting that finding in animal studies could be translated to humans. Using PET/MRI scanners and a marker of glial activation, Loggia et al. (2015) found that glial activation was higher in the thalamus, somatosensory cortex, cingulate cortex, ventromedial prefrontal cortex, and insular cortex in patients suffering from chronic back pain compared with control subjects (Loggia et al., 2015).

Numerous studies have suggested that reactive astrocytes in the spinal cord are associated with chronic pain. Astrocyte activation, indicated by GFAP upregulation, is reported in various pain models (Garrison et al., 1991; Raghavendra et al., 2004; Zhuang et al., 2006; Shi et al., 2012). Reducing GFAP in the spinal cord, by intrathecal injection of GFAP antisense oligonucleotide, reduced pain behaviors associated with spinal nerve ligation injury in rats (Kim et al., 2009). Also, direct activation of astrocytes in the spinal cord in naïve mice, using an optogenetic technique, induced symptoms of neuropathic pain such as mechanical and thermal hypersensitivity (Nam et al., 2016), supporting that activating astrocytes alone is enough to generate neuropathic pain-like behaviors.

Astrocytes in the thalamus, part of the discriminative pain pathway, were also found to be reactive under chronic pain conditions. Chronic constriction injury, which causes neuropathic pain, significantly enhanced GFAP expression in the thalamus (Giardini et al., 2017). Spinal nerve ligation, another neuropathic pain model, also increased the GFAP-immunostained area in the ventral posterolateral thalamus (VPL), while the number of GFAP/S100b+ astrocytes remained unchanged (Blaszczyk et al., 2018). This indicates that astrocytes in VPL increased their volume, indicative of hypertrophy, while their number did not change (no astrocyte proliferation). However, another study that investigated supra-spinal astrocyte reactivity, using the spared nerve injury model, did not find any differences in GFAP expression in the thalamus (Marcello et al., 2013). This discrepancy may be due to a slight difference in pain models used or method of analysis. The involvement of thalamic astrocytes in chronic pain is less clear than reactive microglia in the thalamus, which are consistently found, but a human study suggested that reactive astrocytes in the thalamus may have clinical implications. Patients with neuropathic pain associated with aberrant thalamocortical rhythms (thalamocortical dysrhythmia) exhibited brain rhythms that oscillated in a frequency similar to the frequency of Ca^2+^ waves displayed by activated astrocytes (<0.1 Hz), suggesting that reactive astrocytes in the thalamus may be the underlying cause (Alshelh et al., 2016). Accordingly, treatment with palmitoylethanolamide reduced glial reactivity and reduced oscillatory activity in the pain pathway only in patients who experienced pain reduction (Alshelh et al., 2019).

The activity of astrocytes in the primary somatosensory cortex (S1), the region important for identifying the intensity and location of pain, is also enhanced by nerve injury. GFAP expression and frequency of astrocytic Ca^2+^ activity of astrocytes were enhanced in S1 (Kim et al., 2016; Ishikawa et al., 2018). Increased astrocytic Ca^2+^ activity in S1 after nerve injury was reportedly due to the resurgence in expression of the metabotropic Glu receptor 5 (mGluR5; Kim et al., 2016), which is almost absent in adult mice and humans under physiological conditions (Sun et al., 2013). mGluR5 activates IP3R, which leads to Ca^2+^ release from the ER. Inhibiting the resurgence of mGluR5 reduced allodynia while enhancing mGluR5 expression induced allodynia in naïve mice (Kim et al., 2016), strongly supporting that increased astrocytic Ca^2+^ signals by mGluR5 trigger chronic pain symptoms. Consistently, inhibiting S1 astrocyte metabolism with fluoroacetate, which also blocks astrocytic Ca^2+^ activity, reduced mirror pain (pain occurring in the unflicked contralateral side) resulting from nerve injury (Ishikawa et al., 2018).

Astrocytes in brain regions known to process the cognitive-affective aspect of pain are also activated with chronic pain. Sciatic nerve ligation significantly enhanced GFAP expression in the cingulate cortex (Kuzumaki et al., 2007; Yamashita et al., 2014). Injection of Complete Freund’s Adjuvant (CFA), which induces chronic inflammatory pain, also enhanced GFAP expression in the ACC bilaterally (Chen et al., 2012). Interestingly, L-alpha-aminoadipate (astrocyte toxin) injection into the ACC inhibited place escape/avoidance behavior, which measures aversive emotion associated with nociceptive stimuli, but paw withdrawal threshold was unaffected, suggesting that astrocyte activation in the ACC only regulates the affective component of pain (Chen et al., 2012). GFAP expression was also increased in the amygdala (brain structure implicated in processing negative emotion) and the medial prefrontal cortex (structure implicated in cognitive processing; Alvarado et al., 2013; Marcello et al., 2013). Enhanced GFAP expression is also reported in the hippocampus (structure important for learning and memory; Zhu et al., 2009).

The mechanism underlying the activation of astrocytes along the pain processing pathway—structures not directly affected by injury—during chronic pain remains unknown. One possible mechanism could be through the propagation of Ca^2+^ waves between astrocytes connected by gap junctions. As described in the “Introduction” section, reactive astrocytes exhibit prominent Ca^2+^ waves, which enable long-distance communication. Astrocytes are physically connected *via* gap junctions, composed mainly of connexin (Cx) subtype Cx43 (Nagy et al., 1999). Ca^2+^ waves propagate by diffusion of IP3 through gap junctions (short distance signaling) or diffusion of extracellular ATP (long-distance signaling; Fujii et al., 2017). Connexins could also act as hemichannels releasing various molecules such as ATP, Glu, and chemokines, which can contribute to astrocyte activation. Cx43 is upregulated in reactive astrocytes and implicated to have important roles in chronic pain development and maintenance (Xing et al., 2019). In particular, mirror pain induced by nerve injury or inflammation was reduced by carbenoxolone, a non-selective gap junction inhibitor (Spataro et al., 2004; Choi et al., 2017), suggesting that astrocytic gap junctions could contribute to astrocyte activation along the pain pathway. However, Cx43 alone may not regulate pain sensitivity, as suggested by a recent finding that downregulation of spinal astrocytic Cx43 induced mechanical hypersensitivity in naïve mice (Morioka et al., 2018).

## Relationship Between Astrocytic Calcium and Pain

While some studies suggest that aberrant changes in astrocytic Ca^2+^ underlie pathological pain, detailed mechanisms remain to be determined. Generally, increased astrocytic Ca^2+^ has been associated with increased pain, while reduced astrocytic Ca^2+^ has been associated with reduced pain. We can begin to understand these mechanisms by taking into consideration the various sources and consequences of astrocytic Ca^2+^ changes (as described in “Source of Calcium in Astrocytes” section above).

Astrocytic Ca^2+^ increased in the S1 and the ACC following nerve injury (Yamashita et al., 2014; Kim et al., 2016; Ishikawa et al., 2018). Astrocytic Ca^2+^ changes were also shown to be associated with synaptogenic thrombospondin (TSP-1) release and synapse formation, indicating that rewired circuits could cause neuropathic pain (Kim et al., 2016). Directly activating spinal astrocytes with optogenetic [channelrhodopsin-2 (ChR2)] stimulation, which increased astrocytic Ca^2+^, also triggered mechanical and thermal hypersensitivity in naïve animals (Nam et al., 2016), much like that seen in animals inflicted with chronic pain, suggesting that an increase in astrocytic Ca^2+^ can contribute to the induction of pain. However, since ChR2 activation of spinal astrocytes also induced the release of ATP and pro-inflammatory mediators along with enhanced GFAP expression (Nam et al., 2016), enhanced astrocytic Ca^2+^ alone may not be enough to induce pain hypersensitivity. An increase in Ca^2+^ waves generated by reactive astrocytes is also suggested to contribute to pathological pain symptoms in patients with neuropathic pain (Alshelh et al., 2016).

Conversely, reduction in astrocytic Ca^2+^ has been associated with attenuated pain responses. Blocking astrocytic Ca^2+^ with fluoroacetate, astrocyte metabolism inhibitor, reduced mirror pain responses that accompany neuropathic pain (Ishikawa et al., 2018). IP3R2 knockout mice, which have significantly reduced astrocytic Ca^2+^ signals, especially the prominent astrocytic Ca^2+^ signals in the cell body as discussed in “Source of Calcium in Astrocytes” section, also displayed reduced mirror pain following nerve injury (Ishikawa et al., 2018).

Up until now, only a few studies have investigated the relationship between astrocytic Ca^2+^ and chronic pain, but these studies consistently suggest that a global, not local, increase in astrocytic Ca^2+^ may cause chronic pain. Activation of both the IP3R and the ChR2 induce global Ca^2+^ change in astrocytes. The specific type of Ca^2+^ channels involved in ChR-2 mediated changes in astrocytic Ca^2+^ is uncertain. However given that ChR2 is a light-activated non-selective cation channel, ChR2 activation will have induced Ca^2+^ influx, along with other cations, from extracellular space. Influx, or efflux, of cations *via* ChR2 may further activate other Ca^2+^ channels that could increase astrocytic Ca^2+^, leading into a chain reaction of intracellular Ca^2+^ increase. Spreading Ca^2+^ increase between astrocytes by ChR2 activation may also contribute to the neuropathic pain-like symptoms since photo-stimulation of astrocytes also released ATP, a molecule important for generating astrocytic Ca^2+^ waves. Furthermore, photo-stimulation of the ChR2 expressing astrocyte increases intracellular Ca^2+^ even in astrocytes that do not express ChR2, reportedly *via* NMDA receptor activation (Berlinguer-Palmini et al., 2014). These studies support the possibility that propagation of Ca^2+^ waves between astrocytes by photo-stimulation could have caused pro-inflammatory mediator release and enhanced GFAP expression, which eventually could have caused neuropathic pain-like symptoms in naïve mice.

How the increase in astrocytic Ca^2+^ leads to chronic pain is unclear, but changes in the interaction between neuron–astrocyte may be one factor. Kim et al. (2016) showed that astrocytic Ca^2+^ changes regulate the release of the synaptogenic molecule thrombospondin (TSP-1), which remodel synapses. Altered connectivity between neurons and associated hyperactivity may be the underlying cause of chronic pain. Since reactive astrocyte exhibits prominent Ca^2+^-dependent gliotransmitter release, enhanced Glu released by reactive astrocytes that excessively excite neurons may also contribute to chronic pain. Changes in neurotransmitter buffering activity could also contribute to the changes in neuron–astrocyte interaction ensuing chronic pain. Buffering of the inhibitory transmitter—GABA—by astrocytes *via* GAT is directly affected by astrocytic Ca^2+^, with more GABA taken up with increasing Ca^2+^. Although GAT activity was shown to enhance the synaptic efficacy of an inhibitory synapse by reducing ambient GABA that desensitize the inhibitory synapse (Shigetomi et al., 2011), this condition may not apply to reactive astrocytes. Increased internal Ca^2+^ of reactive astrocytes may greatly reduce the synaptic GABA concentration to a level that reduces the efficacy of inhibitory synapses.

The release of inflammatory mediators by elevated astrocytic Ca^2+^ may also contribute to chronic pain symptoms. Activation of STIM1 and Orai1 channels, which are major channels for Ca^2+^ influx from the extracellular space, promoted the production of pro-inflammatory mediators such as TNFα and IL-6 in spinal astrocytes (Gao et al., 2016). The secretion of IL-6 was also shown to be regulated by astrocytic Ca^2+^ (Codeluppi et al., 2014). Inflammatory mediators are well-known to enhance astrocytic Ca^2+^ (Agulhon et al., 2012) and will further amplify astrocytic Ca^2+^ signaling. Overall, these studies suggest that astrocytic Ca^2+^ and chronic pain have a close interrelationship, but further studies need to be carried out to reveal more detailed mechanisms. Multiple interrelated events triggered by astrocytic Ca^2+^, some occurring sequentially as astrocytic cytokine expression precedes increased GFAP expression (Norden et al., 2016), will likely contribute to the development of chronic pain. Although few studies have directly investigated the relationship between astrocytic Ca^2+^ and pain, numerous studies report that various channels, discussed in “Source of Calcium in Astrocytes” section, contribute to an increase in astrocytic Ca^2+^ after injury or inflammation, conditions that could cause pain. Other than the IP3R, which was shown to be related to chronic pain, RyR3, STIM1, Orai1, TRPs, and VGCCs also have been implicated in injury/inflammation and ensuing astrocyte activation. Since astrocytic Ca^2+^ signals induced by different channels have different effects and functions, it would be interesting to investigate how each of these channels differentially contributes to chronic pain.

## Conclusion

Astrocytic Ca^2+^ is integral in maintaining CNS function. Following an injury, astrocytes become reactive and go under morphological, chemical, and functional changes. Astrocytic Ca^2+^ dynamics also change with injury, inflammation, or pain. Astrocyte Ca^2+^ channels shown to increase expression/activity with inflammation or injury include IP3R, Orai, Ca^2+^-permeable TRP channels, and VGCCs. Gap junction channels are also Ca^2+^ permeable, and Cx43 was reported to be upregulated with injury, but the diffusion of Ca^2+^ through gap junctions is slow and their contribution to Ca^2+^ signaling would be relatively small. These changes all enhance intracellular astrocytic Ca^2+^ concentration, so an increase in astrocytic Ca^2+^ appears to underlie pathological pain. However, it is not clear what potentiated astrocytic Ca^2+^ signal does to cause pathological pain. Does it act to protect or damage the CNS? The answer may not be clear-cut, as in the example of glial scars. Glial scars were viewed as an obstacle to functional recovery after damage because it hinders axon regeneration; however, preventing glial scar formation results in greater damage (Sofroniew, 2015). Likewise, potentiated astrocytic Ca^2+^ will likely have both protective and detrimental effects. On the other hand, different types of reactive astrocytes, the A1 (neurotoxic) and the A2 (neuroprotective; Liddelow and Barres, 2017; Liddelow et al., 2017), may have different effects on chronic pain. Although the presence or contribution of A2 reactive astrocytes to chronic pain is not yet validated, the protective effect neuroprotective effect exerted by reactive astrocytes after an injury is speculated to be attributed by the A2 subtype, which are induced following injuries such as ischemia (Li et al., 2019). Whether and how the two different types of reactive astrocytes lead to distinct changes in astrocytic Ca^2+^ is unknown. It is possible that astrocytic Ca^2+^ induced by different channels may drive reactive astrocytes to be one type or the other. Therefore, investigating how different astrocytic Ca^2+^ channels contribute to different types of reactive astrocytes in regard to chronic pain development will provide a deeper understanding of the roles of astrocyte in pain and may offer better targets for pain control.

## Author Contributions

JC and YH contributed equally to the manuscript. All authors contributed to the article and approved the submitted version.

## Conflict of Interest

The authors declare that the research was conducted in the absence of any commercial or financial relationships that could be construed as a potential conflict of interest.
